# Energy, the driving force behind good and ill health

**DOI:** 10.3389/fcell.2014.00028

**Published:** 2014-07-11

**Authors:** Vasu D. Appanna, Christopher Auger, Joseph Lemire

**Affiliations:** ^1^Department of Chemistry and Biochemistry, Faculty of Science and Engineering, Laurentian UniversitySudbury, ON, Canada; ^2^Department of Biological Sciences, University of CalgaryCalgary, AB, Canada

**Keywords:** mitochondria, energy metabolism, metabolism, enzymology, bioenergetics

As our toolset to probe the inner workings of biological systems evolves, so too does our understanding of the molecular mechanisms involved in disease processes. Thus, the importance of energy metabolism in the pathology of a variety of disorders is only now beginning to come to light. To that end, a perturbation in bioenergetics has been attributed as a major contributing factor to such conditions as aging and carcinogenesis. This is not surprising since biochemical energy is required for replication, protein synthesis/turnover, membrane production, import/export processes, cell signaling, cellular trafficking, and a myriad of other bodily functions. It stands to reason then, that the balance of energy production and consumption is the fulcrum to maintaining not only cellular homeostasis, but also the well-being of the whole organism. As such, the status of metabolic pathways that participate in energy production can be a strong predictor for diseases. Indeed, recent molecular, biochemical, and physiological studies tend to support the hypothesis that cellular energy status is a determining factor for multiple disorders (DeBerardinis and Thompson, 2012).

The energy status of the cell orchestrates an extensive, highly conserved regulatory network that balances all aspects of cellular function. Indeed, AMP-activated protein kinase (AMPK), a molecular switch that activates multiple signaling pathways in response to cellular energy levels, plays a crucial role in energy sensing and maintaining cellular homeostasis. When energy levels fall within the cell (AMP/ADP:ATP ratio increases), AMPK activates, and coordinates cellular activities in a concerted effort to reduce ATP usage and increase energy production. This includes, but is not limited to decreasing protein synthesis, lipogenesis, fatty acid production, nucleotide synthesis, and gluconeogenesis coupled to an increase in mitochondrial biogenesis, glycolysis, glucose uptake, and autophagy. It is not surprising then that deregulation of AMPK has been implicated in uncontrolled cell division and many disease states such as neurodegeneration, diabetes, and inflammation (Burkewitz et al., 2014). Although experimental evidence suggests that AMPK may be the conductor of cellular events in response to cellular energy status, many other players partake in the orchestration. Peroxisome proliferator-activated receptor gamma coactivator 1-alpha (PGC-1α), phosphatase and tensin homolog (PTEN), uncoupling proteins (UCPs), and sirtuins (SIRT1) that directly or indirectly respond to cellular energy content are some of the effectors. These energy regulators have been the focus of efforts to develop anti-aging therapeutics and treatments for various disease states such as cancer, neurodegeneration, and diabetes (Ortega-Molina and Serrano, 2013). The importance of these moieties is highlighted by the pivotal position they hold in the crosstalk between cellular energy status and proper cell functioning.

The necessity of maintaining a balanced cellular energy status for effective physiological functions places the mitochondrion at the center of health and disease. Indeed, this organelle is the energy powerhouse of the cell, creating the majority of the ATP used for cellular operation. Recently, the disturbance of mitochondrial function has been underscored as a key feature in the progress of degenerative disorders. For example, genetic mutations in genes that encode for mitochondrial machinery have been proposed as a hallmark of age-related degenerative disorders (Wallace, 2013). Moreover, the mitochondrion is thought to be at the root of insulin resistance, metabolic syndrome, cardiovascular disease, and cancer (Ren et al., 2010; Rocha et al., 2014). The importance of this compartment is amplified in energy-intensive organs such as the heart, brain, and muscle tissue. As such, it is not surprising that mitochondrial dysfunction and ineffective ATP synthesis have been linked to a range of disorders that include cardiac arrhythmias, Parkinson's disease, obesity, muscle atrophy, and infertility (Da Cruz et al., 2012; Wallace, 2013; Youdim and Oh, 2013). To that end, rejuvenating the capacity of the mitochondria to produce energy, increasing mitochondrial biogenesis as well as the administration of mitochondria-targeted antioxidants has received credence as potential therapeutic techniques to alleviate some disorders. Indeed, the tricarboxylic acid (TCA) cycle and oxidative phosphorylation, the two key mediators of ATP production play a critical role in the health of the mitochondria and hence of the bodily functions. The TCA cycle does not only act as a provider of reducing factors, ingredients that drive ATP synthesis but also generates metabolites designed to act as anti-oxidants and regulators of aerobic respiration. In fact, succinate is known to promote glycolytic ATP formation, a feature utilized by rapidly dividing tumors. The molecular machinations of these ATP-generating networks may reveal the secrets underlying good and ill health (Mailloux et al., 2007).

Hence, maintaining a balanced energy status in the cell is critical and is orchestrated by elaborate signaling programs linked to an intricate feedback regulation of these energy-producing pathways. This tightly governed metabolic balancing act is centered on the mitochondrion, the energy generator of the cell. Indeed, bioenergetics is at the crux of cellular function, and the dysregulation of energy metabolism holds the key to the unraveling of disease conditions (Figure 1). Understanding the molecular pathways aimed at the generation and utilization of energy coupled with their regulation will help unearth various cellular malfunctions and pave the way to develop cures against various maladies.

**Figure 1:**
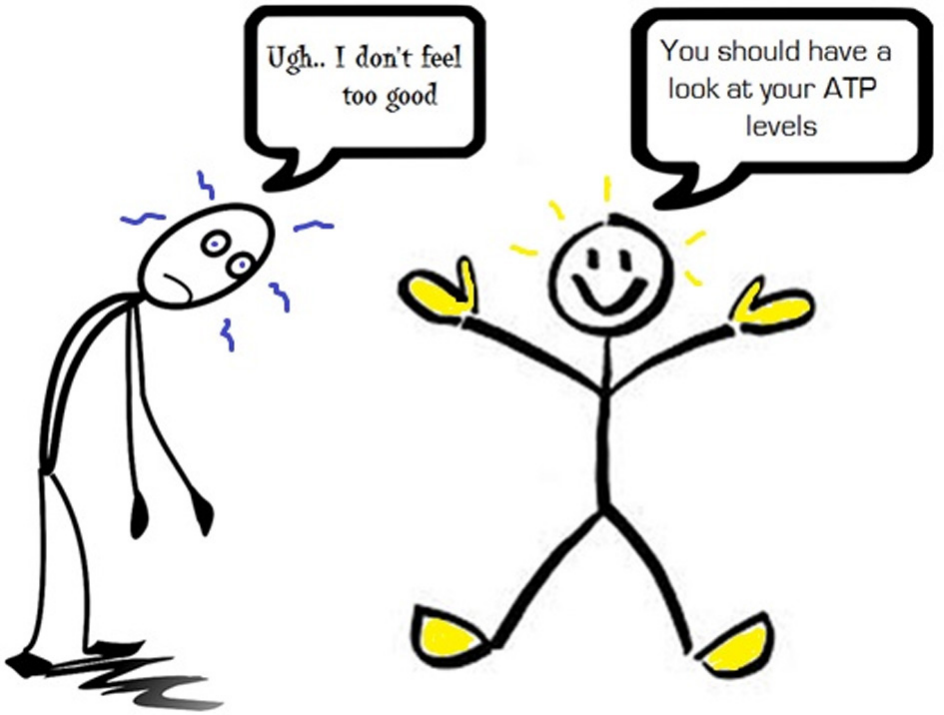
**ATP: The molecular key to good health**.

## Conflict of interest statement

The authors declare that the research was conducted in the absence of any commercial or financial relationships that could be construed as a potential conflict of interest.
